# M1 and M2 Functional Imprinting of Primary Microglia: Role of P2X7 Activation and miR-125b

**DOI:** 10.1155/2016/2989548

**Published:** 2016-12-20

**Authors:** Chiara Parisi, Giulia Napoli, Pablo Pelegrin, Cinzia Volonté

**Affiliations:** ^1^CNR-Institute of Cell Biology and Neurobiology, Via del Fosso di Fiorano 65, 00143 Rome, Italy; ^2^Fondazione Santa Lucia, Via del Fosso di Fiorano 65, 00143 Rome, Italy; ^3^Inflammation and Experimental Surgery Unit, CIBERehd, Biomedical Research Institute of Murcia (IMIB-Arrixaca), Clinical University Hospital Virgen de la Arrixaca, 30120 Murcia, Spain

## Abstract

Amyotrophic lateral sclerosis (ALS) is a most frequently occurring and severe form of motor neuron disease, causing death within 3–5 years from diagnosis and with a worldwide incidence of about 2 per 100,000 person-years. Mutations in over twenty genes associated with familial forms of ALS have provided insights into the mechanisms leading to motor neuron death. Moreover, mutations in two RNA binding proteins, TAR DNA binding protein 43 and fused in sarcoma, have raised the intriguing possibility that perturbations of RNA metabolism, including that of the small endogenous RNA molecules that repress target genes at the posttranscriptional level, that is, microRNAs, may contribute to disease pathogenesis. At present, the mechanisms by which microglia actively participate to both toxic and neuroprotective actions in ALS constitute an important matter of research. Among the pathways involved in ALS-altered microglia responses, in previous works we have uncovered the hyperactivation of P2X7 receptor by extracellular ATP and the overexpression of miR-125b, both leading to uncontrolled toxic M1 reactions. In order to shed further light on the complexity of these processes, in this short review we will describe the M1/M2 functional imprinting of primary microglia and a role played by P2X7 and miR-125b in ALS microglia activation.

## 1. Introductory Statement

The hardest challenge in microglia research is to decode a metamorphosis between the two ramified/surveilling and amoeboid/activated morphologic-functional end points and to establish a biological link between two forms of power, the anti- and the proinflammatory one. To this aim, molecular information becomes the master key to direct what has to be done in microglia, in other words if and how to respond to an internal or external stimulus with a consequent either aggressive inflammatory or compliant anti-inflammatory behavior. Along the flow of information that step by step instructs microglia on the “right” thing to do, understanding how the activation of a receptor sensing the environment translates an extracellular stimulus into an intracellular signal surely seems important. Under this regard, the P2X7 receptor activated by extracellular ATP possesses a privileged position. Extracellular ATP is not only a primordial precursor in the evolution of the chemical transmission and an ubiquitous intercellular communication agent, but also the chief endogenous microglia alarm signal. In addition, P2X7 is highly expressed in microglia, is involved in inflammatory responses, and possesses a dual either trophic and anti-inflammatory or toxic and proinflammatory function. In the regulation of microglial P2X7 by extracellular ATP, microRNAs, among which particularly miR-125b, are lately emerging as key modulators of receptor signaling and functional microglia commitment under basal or amyotrophic lateral sclerosis- (ALS-) evoked inflammatory conditions. In this work, we will describe the M1/M2 functional imprinting of primary microglia as a paradigm of pro/anti-inflammatory function and the role played by P2X7 and miR-125b in ALS microglia activation.

## 2. Basic Features of ALS

The two-century-old rare disease ALS is the most frequently occurring and severe form of progressive upper and lower motor neuron disease, causing death within 3–5 years from diagnosis [1, 2], with a worldwide incidence of ~2 per 100,000 person-years [3] and without any curative treatment to date. Mutations within the superoxide dismutase 1 (*SOD1*) gene coding for the ubiquitously expressed enzyme SOD1 [4–6] are responsible for about a quarter of the inherited disease cases. Additional mutations in over twenty genes, among which the most frequent C9ORF72, TAR DNA binding protein 43 (TARDPB), and fused in sarcoma (FUS), have been associated with familial forms of ALS and account for about 10–15% of all ALS cases, providing insights into the complex genetic mechanisms that lead to motor neuron death [6]. Notably, mice that express mutant human SOD1 protein recapitulate most of the key features of ALS, comprising motor neuron degeneration and decreased life span, and are the most exploited animal model for studying the disease pathogenesis [7].

The hallmarks of ALS include genomic instability, epigenetic alterations, cellular senescence, stem cell exhaustion with decreased tissue regenerative potential, deregulated nutrient-sensing, mitochondrial dysfunction and oxidative stress, loss of proteostasis including autophagy impairment, altered intercellular communication and excitotoxicity, and central neuroinflammation sustained by microglia/macrophages and astrocytes [8]. Most recent advances have moreover recognized that the complicity of all types of glial and inflammatory cells at both peripheral and central level indeed contributes to endangering and compromising the motor neuron function [9, 10]. This in part explains the multigenic, multisystemic, multifactorial, and non-cell-autonomous nature of ALS.

Shared consent to explain the pathogenic mechanisms of ALS includes the release of proinflammatory factors and toxic agents and the prompt activation of glial cells, overall culminating into injury to motor neurons. Because extracellular ATP activating purinergic P2 receptors and particularly the P2X7 subtype [11] constitutes a lead neuron-to-microglia alarm signal, a role for purinergic receptor signaling in ALS neuroinflammation has been highlighted [12, 13]. The P2X7, since its discovery in lymphocytes and mast cells, has been mainly implicated in the processing and release of cytokines such as interleukin-1*β* (IL-1*β*) and involved in neuroinflammation and immunity, as well as in the initiation of cell death mediated by apoptosis, necrosis, and autophagy mechanisms [14]. Recent work provided evidence that modulation of microglia via the P2X7 also constitutes a novel pathway involved in ALS progression [15–19]. Moreover, Brilliant Blue G, a poorly centrally penetrant and low affinity P2X7 antagonist, was shown to reduce microgliosis but not astrocytosis in lumbar spinal cord, to modulate inflammatory genes such as nuclear factor kappa B (NF-kB), NADPH oxidase 2 (NOX2), IL-1*β*, interleukin-10 (IL-10), and brain derived neurotrophic factor (BDNF), to enhance motor neuron survival, and to induce a slightly delayed ALS disease onset with modest improvement of general conditions and motor performance, when provided to SOD1-G93A mice from late preonset [20]. Overall, these findings would suggest that P2X7 might be a susceptibility or modifying target in ALS pathogenesis.

## 3. Plasticity of Microglia

Since their discovery almost a century ago by del Río Hortega, microglia constituting about 10–15% of cells located in the brain and spinal cord are the first and main form of defense in the CNS, being responsible for homeostasis maintenance [21]. To this aim and in contrast to peripheral macrophages, microglia have developed a spatially and temporally tightly regulated mechanism of compliance to the extracellular environment, becoming extremely sensitive even to the smallest physiological perturbation or generic danger signal present in their surrounding area [22]. While the basic steady state of microglia is tolerant, prohomeostatic, and surveilling the environment, on the other hand there is no indication that neuronal damage might occur without activation of microglia. Accordingly, the main response of microglia to neuronal stress is rapid retraction of the fine processes and gradual transition from ramified/surveilling into a more amoeboid macrophage-like phagocytic cell. Only for investigation needs, the complexity of this transition has been simplified and categorized into a stereotypic process with basically two morphological ending phenotypes: ramified/surveilling and amoeboid/activated, often correlated with a migratory behavior and/or proliferation of the cells [23]. Accordingly to a functional scale, microglia activity can be then categorized into a classical proinflammatory and neurotoxic phenotype, known as M1, and the alternative anti-inflammatory M2 phenotype involved in the resolution of inflammation, phagocytosis and tissue repair [24]. These are considered as the two end points of a broad scale of microglia responses, of a continuous spectrum of functional heterogeneity. M1-like microglia generally express IL-1*β*, interleukin-6 (IL-6), tumor necrosis factor *α* (TNF*α*), nitric oxide synthase 2 (NOS2), and CD16/32, whereas M2-like microglia express lower levels of these markers, but higher levels of IL-10, BDNF, Arginase-1 (Arg1), and Mannose Receptor C, type 1 (MRC-1) [25]. Interestingly, the phenotypic identity of microglia is also age-dependent, but what is clear is that these cells are not fully committed to either an M1 or an M2 phenotype neither in newborn nor in adult mice. For instance, in early postnatal CNS, microglia express M1 markers such as iNOS and TNF*α* but also express the M2 marker Arg1 [26]. This pattern changes in old mice and microglia appear to be committed to a stronger M1 phenotype with higher levels of proinflammatory IL-1*β* and IL-6 mRNA than those observed, for instance, at 12 months of age [26]. The participation of microglia to ALS pathogenesis has been thoroughly investigated in sophisticated experiments in which mutated SOD1 was expressed in specific cell phenotypes. The main conclusion that arose from those studies was that several neurotransmitters can control neuroinflammation and neurotoxicity by activated microglia and that mutated SOD1 microglia do not initiate motor neuron degeneration but rather accelerate disease progression [27–29]. In aging SOD1 overexpressing ALS mice, a prevalence of M1-like microglia is indeed observed that is moreover neurotoxic in vitro [30]. However, in vivo/in vitro evidence about microglia being either M1 detrimental or M2 protective that is merely based on immunostaining of morphologic markers and correlation studies is limitative and not standing on a solid ground. Additional information is absolutely required to explain the activation of these cells and particularly their role in the ALS brain.

## 4. Microglia versus Macrophages

Different combinations of inflammatory factors are known to be able to polarize microglia into a variety of activation states whose characterization is however still under scrutiny [31]. For instance, in vitro studies have determined that an environment dominated by proinflammatory stimuli, such as lipopolysaccharide (LPS) and interferon-*γ* (IFN*γ*), favors polarization toward M1 effector cells, while anti-inflammatory cytokines such interleukin-4 (IL-4) enable the M2 protective phenotype [32]. However, there is still a lot to be learned about the molecular mechanisms governing microglia plasticity across these different activation states [33]. In an attempt to understand microglia responsiveness to temporal combinations of M1 and M2 inflammatory stimuli and furthermore to establish potential discriminatory differences between central microglia and peripheral macrophages, we exploited an in vitro protocol based on the responsiveness of peritoneal macrophages to an inflammatory polarization gradient [34, 35]. This protocol generates a gradient of five polarity phenotypes. In particular, proinflammatory stimuli are used to differentiate cells into an M1 phenotype (state 1), while anti-inflammatory stimuli are used to differentiate them into M2 (state 4). A combination of both M1 and M2 stimuli is then used to differentiate microglia into an intermediate M1/M2 polarization phenotype (state 3). Finally, to study the polarity switch between M1 and M2 states, microglia are stimulated firstly with anti-inflammatory stimuli, washed, and then stimulated with proinflammatory ones and vice versa.

**Figure 1:**
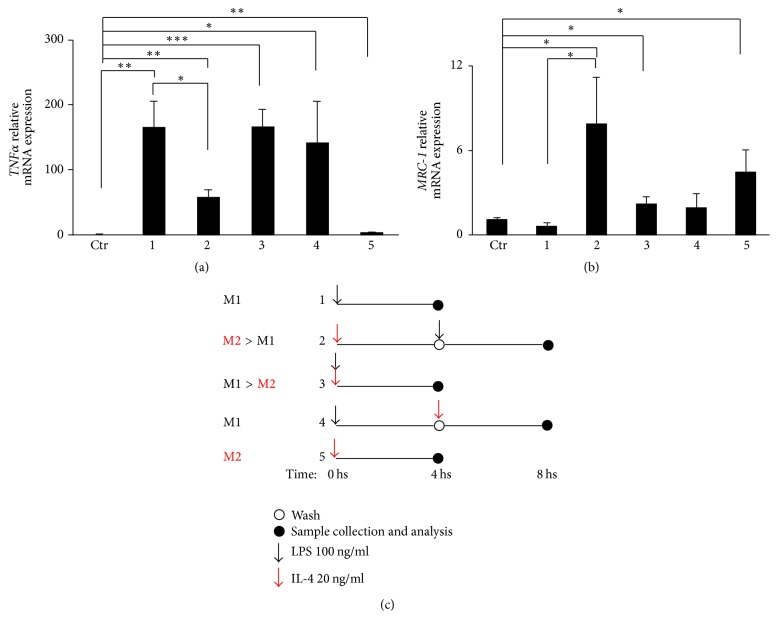
Microglia responsiveness to macrophages polarity gradient. Primary microglia obtained from brain cortex of P0/P1 C57BL/6 mice were prepared as previously described [15]. (a)* TNFα*or (b)* MRC-1* expression levels were measured by qRT-PCR using primer pairs from Qiagen-GeneGlobe program. Data are calculated using the 2^−ΔΔCt^ method and GAPDH transcript as normalizer (Qiagen-GeneGlobe program). Bars represent mean ± sem of ≥3 independent experiments; ^*∗*^ *P* < 0.05, ^*∗∗*^ *P* < 0.01, and ^*∗∗∗*^ *P* < 0.001 versus untreated cells by unpaired *t*-test. (c) Schematic representation of the response of primary microglia to polarity gradient protocol used as revised from [34].

Similarly to peritoneal macrophages, we report here that primary microglia can polarize into M1-like phenotype characterized by high TNF*α* and low MRC-1 expression, when exposed to the inflammatory stimuli LPS alone (M1/state 1, Figures 1(a)–1(c)). Likewise, microglia polarize toward an M2-like phenotype characterized by low TNF*α* and high MRC-1 levels, when exposed to the anti-inflammatory cytokine IL-4 alone (M2/state 5, Figures 1(a)–1(c)). Surprisingly and unlike peripheral macrophages that are able to reversibly and dynamically swing among different activation states in vitro [34], the addition of either LPS after IL-4 washout (M2 > M1/state 2, Figures 1(a)–1(c)) or IL-4 after LPS washout (M1/state 4, Figures 1(a)–1(c)) is not able to counteract the M2 (low TNF*α* and high MRC-1 expressions) or M1 (high TNF*α* and low MRC-1 expressions) commitment initially elicited by microglia in vitro. Finally, the simultaneous addition of proinflammatory LPS and anti-inflammatory IL-4 (M1 > M2/state 3, Figures 1(a)–1(c)) directs microglia toward a preponderant M1 phenotype, while this same condition is known to induce hybrid M1/M2 phenotype in peritoneal macrophages [34]. Therefore, different from peripheral macrophages where an initially induced M1 or M2 phenotype can be reversibly and, respectively, redirected toward an M2 or M1 activation state, respectively, by IL-4 or LPS [34], primary microglia undergo an irreversible either pro- or anti-inflammatory “imprinting” once they are firstly challenged with, respectively, M1 or M2 inflammatory stimuli. In other words, central microglia appear less prone than peripheral macrophages to phenotypic redirection. These findings are in line with previous results indicating that stimulation of microglia with IL-4 prior to LPS prevents the LPS-mediated inhibition of the microglial neuroprotective effects and the release of neurotoxic factors [36, 37]. Moreover, they raise questions about the dynamics and versatility of microglia engagement into a specific functional phenotype, and most importantly they might contribute to explaining why microglia-targeted anti-inflammatory therapy has failed in ALS so far.

## 5. MiRNA-125b in ALS

MicroRNAs (miRNAs), endogenous small RNA molecules that have emerged as key regulators of target gene expression at the posttranscriptional level [38], behave as fine-tuners in controlling diverse biological processes at the molecular, cellular, and tissue level, including brain functioning. In the CNS, as much as 30% difference in miRNA expression profile exists among neurons, oligodendrocytes, astrocytes and microglia, thus explaining why cell-specific modulations of miRNA levels can easily contribute to orchestrating the complex network of interactions that regulate brain functioning [39]. In particular, miRNAs are also good candidates for distinguishing for instance brain resident microglia from infiltrating monocytes/macrophages or the different activation states of microglia [40, 41] thus becoming potential biomarkers and molecular targets during neuroinflammation and neurodegeneration. Not surprisingly, miRNAs dysregulations are well established also in ALS [42, 43] and in the last few years several works have demonstrated changes in the expression of selected miRNAs that are associated with both familial and sporadic cases [44–53]. In addition, studies performed with the SOD1-G93A mouse model have shed light on the role of specific miRNAs in ALS [52–54] and opened miRNA targeting as a new conceivable opportunity for ALS treatment [54–56].

In previous work, we have compared miRNAs transcriptional profiling of nontransgenic and ALS microglia under resting conditions and after inflammatory activation induced by extracellular ATP through P2X7 receptor, by identifying the upregulation of immune-enriched miR-22, miR-155, miR-125b, and miR-146b in ALS microglia. Moreover, we proved that miR-125b increases TNF*α* transcription by interfering with the STAT3 pathway. As in turn TNF*α* upregulates miR-125b and inhibitors of miR-125b reduce TNF*α* expression levels, we recognized the induction of miR-125b as a vicious gateway culminating in abnormal TNF*α* release [18]. Moreover, we have established an interplay between miR-125b and A20 protein in the modulation of classical NF-*κ*B signaling sustained in microglia. In ALS, classical NF-*κ*B pathway is indeed known to be related to persistent microglia activation and motor neuron injury [57] but mechanisms of negative control of NF-*κ*B activity still remain unexplored. One of the major players in the termination of classical NF-*κ*B pathway is the ubiquitin-editing enzyme A20, which has recognized anti-inflammatory functions. In particular, our previous work has highlighted that miR-125b by terminating A20 activity strengthens and prolongs the noxious activation of NF-*κ*B induced by stimulation of P2X7, with deleterious consequences on motor neuron survival. Consistently, miR-125b inhibition by restoring A20 levels can protect motor neuron [19]. Overall, these results strengthen the impact of miRNAs in modulating inflammatory genes linked to ALS and identify miR-125b as a key mediator of microglia dynamics and pathogenic mechanisms in the disease.

### 5.1. P2X7- and miR-125b-Dependent Regulation of Proinflammatory and Anti-Inflammatory Mediators in ALS Microglia

**Figure 2:**
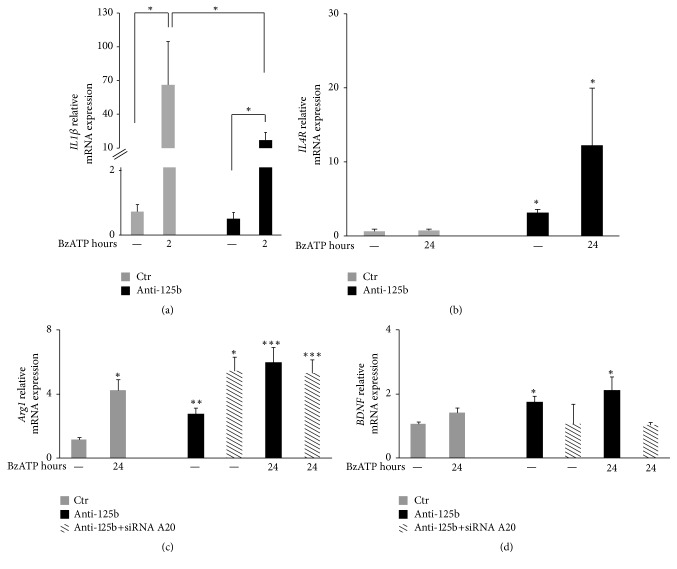
Regulation of proinflammatory and anti-inflammatory mediators by P2X7 activation and miR-125b inhibition in SOD1-G93A microglia. SOD1-G93A primary microglia obtained from brain cortex of P0/P1 B6.Cg-Tg(SOD1-G93A)1Gur/J mice were prepared as previously described [15]. Then, 5 × 10^5^ cells/well were plated and transfected with 20 nM scramble miRIDIAN hairpin inhibitor (ctr) or miR-125b miRIDIAN hairpin inhibitor (anti-125b) for 48 h and exposed to BzATP 100 *μ*M for the indicated times. Expression levels of* IL1β* (a) (primer pairs: F 5′-GCAACTGTTCCTGAACTCAACT-3′; R 5′-ATCTTTTGGGGTCCGTCAACT-3′),* IL4r *(b) (F 5′-CGAGTTCTCTGAAAACCTC-3′; R 5′-CCATCTGGTATCTGTCTG-3′),* Arg1* (c) (F 5′-CCACGGTCTGTGGGGAAAGCCAAT-3′; R 5′-CTGCCAGACTGTGGTCTCCACCCA-3′), and* BDNF *(d) (F 5′-CGGCGCCCATGAAAGAAGTA-3′; R 5′-AGACCTCTCGAACCTGCCCT-3′) were measured by qRT-PCR. Data are calculated using the 2^−ΔΔCt^ method and GAPDH transcript as normalizer (F 5′-CATGGCCTTCCGTGTTCCTA-3′; R 5′-CCTGCTTCACCACCTTCTTGAT-3′). Bars represent mean ± sem of ≥3 independent experiments ^*∗*^ *P* < 0.05, ^*∗∗*^ *P* < 0.01, and ^*∗∗∗*^ *P* < 0.001 versus untreated cells, by unpaired *t*-test.

Several dangerous signals and endogenous triggers, among which extracellular ATP acting on P2X7, are known to contribute to the pathogenic proinflammatory response of ALS microglia. In particular, P2X7 was found increased in activated microglia of ALS patients [58] and its selective activation by the agonist 2′-3′-O-(benzoyl-benzoyl) ATP (BzATP) in primary microglia isolated from SOD1-G93A mouse brain induces morphologic transition, enhances the production of proinflammatory mediators such as TNF*α*, and influences cyclooxygenase-2 (COX2), NOX2, Rac1, ERK1/2, p38, and NF-kB activation [15, 16, 19]. In order to widen our knowledge about the role of P2X7 in ALS neuroinflammation and the suppressive role exerted by miR-125b inhibition on proinflammatory marker production and consequent improvement of motor neuron survival [18, 19], we analyzed the expression of* IL1β* gene transcription after BzATP challenge. The expression of IL1*β* mRNA that is time-dependent and maximally induced by BzATP in two hours (data not shown) is significantly reduced in the presence of miR-125b inhibitor (Figure 2(a)). These data are in line with our previous results on* TNFα* and* NOX2* expression showing that the maximal repressive effect of miR-125b inhibitor is obtained at the peak of induction of these same proinflammatory M1 markers [19].* IL1β*, together with TNF*α*,* NOX2*, and several other genes are known NF-kB responsive genes [59]. Therefore, as for TNF*α*, we do not exclude that regulation of* IL1β* expression by miR-125b might depend on the regulation of the NF-kB canonical pathway by miR-125b that was previously established, for instance, in microglia [19] and in lymphoma B-cells [60].

Due to the phenotypic complexity of microglia, the M1/M2 paradigm is simply assumed as a conventional generalized model to define functional phenotypes, however with the limitation that several markers classified as specific for characterizing M1 or M2 states can instead be found modulated by both M1 and M2 stimuli. For instance, despite the fact that the expressions of the cytokine IL-10 and interleukin-4 receptor (IL-4R) are considered a hallmark of microglia anti-inflammatory M2 phenotype, IL-10 and IL-4R can also be induced by proinflammatory stimuli such as LPS, perhaps as a feedback mechanism to restrain an exuberant immune response [36, 61]. In order to gain further insight into the microglia response to P2X7 activation and to deeper characterize the role of miR-125b on microglia activation, we evaluated the expression of M2 markers in SOD1-G93A microglia.* IL-4R* gene expression is known to be fundamental for microglia responsiveness to IL-4 and conversion toward a motor neuron protective/M2 phenotype [62]. We thus measured IL-4R mRNA levels in the presence of BzATP and with or without miR-125b inhibition. Our results (Figure 2(b)) demonstrates that while the content of IL-4R mRNA is almost undetectable in SOD1-G93A microglia and this occurs independently from BzATP treatment, the presence of miR-125b inhibitor is able to up regulate both constitutive and BzATP-challenged IL-4R levels. Under these same conditions, we surprisingly show that BzATP enhances the expression of Arg1 mRNA levels after 24 h, while miR-125b inhibition again significantly upregulates Arg1 mRNA in the presence or absence of BzATP (Figure 2(c)). This is confirmed also in wild type cells (data not shown). Consistently with previous findings on the induction of IL-4R by LPS [61], we thus demonstrate that also a proinflammatory stimulus such as BzATP is able to induce the content of a typical M2 marker in ALS microglia. However, LPS and BzATP can apparently discriminate among different M2 mediators. Moreover, while BzATP fails in stimulating BDNF mRNA levels in SOD1-G93A microglia, miR-125b inhibition is able to significantly up regulate also BDNF, in the presence or absence of BzATP (Figure 2(d)). These results might further explain the beneficial effects exerted by miR-125b inhibition in SOD1-G93A microglia and motor neuron survival, thus suggesting that the neuroprotective effect exerted by miR-125b inhibition might depend not only on the suppression of toxic mediators among which IL-1*β* (Figure 2(a)), TNF*α*, and NOX2 [19], but also on the direct stimulation of M2 parameters such as IL4R, Arg1, and BDNF in ALS microglia.

## 6. Induction of BDNF by miR-125b Inhibition Is A20-Dependent

In the complexity of microglia responses, several transcription factors among which NF-kB are involved in both M1 and M2 regulation [63]. The NF-*κ*B family of transcription factors consists of five proteins, p65 (RelA), RelB, c-Rel, p105/p50 (NF-*κ*B1), and p100/52 (NF-*κ*B2) whose combination to form distinct transcriptionally active homo- and heterodimeric complexes creates an elaborate system to control the inflammatory response [64, 65]. While the p65/p50 dimer is recognized as a master activator of M1 genes, the accumulation of NF*κ*B p50 and the formation of transcriptionally regulatory p50 homodimers appear to play an important role in the resolution of inflammation through the enhancement of M2 factors [66–71]. Moreover, while the role of p52/RelB dimer resulting from the activation of the so-called noncanonical way remains elusive, studies with RelB-deficient mice and the ability of p52 to bind the IL-10 promoter have also revealed an anti-inflammatory role for this pathway [57, 72, 73]. A20 protein is a master negative feedback regulator of canonical NF-kB signaling [74, 75] and it was recently highlighted as a broader regulator of NF-kB family, due to its positive action also on the noncanonical pathway [76]. As the BDNF promoter has functioning sites recognized for NF-kB family members [77] and miR-125b was reported to regulate A20 protein in SOD1-G93A microglia [19], we then asked if the mechanism by which miR-125b inhibition enhances M2 markers and particularly BDNF and Arg1 could be dependent on A20 acting on NF-kB components. As Figure 2(d) shows, in the absence of A20 we find that miR-125b inhibition fails to stimulate BDNF (but not Arg1) production, thus suggesting the existence of a potential miR-125b-A20-NF-kB-BDNF axis. By reinforcing this hypothesis, the silencing of A20 does not revert the overexpression of Arg1 by miR-125b inhibition (Figure 2(c)), perhaps because Arg1 transcription is known not to be regulated by NF-kB. Moreover, the lack of the A20 silencing effect on Arg1 induction would also suggest that overexpression of Arg1 is not related to the protective effect of microglial A20 toward motor neurons. Under this regards, a potential neuroprotective role of P2X7 would be perhaps not mediated by Arg1 expression.

## 7. Conclusions and Perspectives

Despite the fact that microglia are classified as resident macrophages of the CNS with whom they in part share shape and functions, several evidences prompt to discriminate them from peripheral macrophages, for instance, in terms of strength and plasticity in immune regulatory responses. Our work has shown here that brain microglia in primary culture undergo a preferential and irreversible M1 or M2 imprinting, once challenged, respectively, with M1 or M2 inflammatory stimuli, and this feature contributes to distinguishing microglia from macrophages. Moreover we have demonstrated that activation of P2X7 receptor in SOD1-G93A microglia surprisingly non only stimulates common M1 markers but also increases the M2 parameter Arg1 [15, 16, 20]. This would confirm the hypothesis that indeed P2X7 receptor might be involved not only in ALS toxic actions, but also in microglia-dependent neuroprotection [17, 78, 79], thus sustaining the contribution of P2X7 during the resolution of inflammation in macrophages and not only its active part in the release of proinflammatory cytokines [78]. Therefore, we do believe that a better understanding of the molecules and pathways, as well as the timing, responsible for this functional imprinting of microglia might help to select more effective microglia-targeted therapies during diseases. For instance, there is still no effective and enduring treatment against ALS and efficient therapeutic options to prevent pathological ALS sequelae are urgently needed. On one hand, it is now clear that dysregulations of microglia as responses to neuronal damage strongly contribute to ALS pathology and that microglia are involved in the progression of the disease by exacerbating central inflammatory mechanisms among which activation of P2X7. On the other hand, in the last few years several miRNAs emerging as important tools in the fine-tuning regulation of cell behavior have been associated with ALS. Aberrant expression of miRNAs relevant for neuronal function has repeatedly been reported in the spinal fluid, frontal cortex, and serum of familial and sporadic ALS patients, and miRNA activity has been demonstrated to be essential for long-term survival of motor neurons as well as microglia responsiveness. Actually, miRNAs are now recognized as potential therapeutic targets and biomarkers for neurodegeneration and neuroinflammation in ALS, to the point that identifying miRNAs at work in this diseases is more important than ever. In particular, we have also demonstrated here that miR-125b inhibitors stimulate the expression of M2 markers IL4R, Arg1, and BDNF, other than inhibiting M1 parameters as we have established in previous work [18, 19], thus reinforcing the proinflammatory action, for instance, of miR-125b in ALS.

Overall, we can conclude that a subtle equilibrium in the timing and power of proinflammatory versus anti-inflammatory agents can imprint microglia to tip the balance toward toxicity or protection, motor neuron survival, or cell death in ALS. Up to now we have established the active participation of P2X7 and miR-125b in the inflammatory reaction of microglia; further work will tell us exactly when and how to manipulate these same pathways in order to improve their therapeutic perspective against ALS.

## Abbreviations

ALS: Amyotrophic lateral sclerosis
Arg1: Arginase-1
BDNF: Brain derived neurotrophic factor
BzATP: 2′-3′-O-(Benzoyl-benzoyl) ATP
IFN*γ*: Interferon-*γ*
IL-1*β*: Interleukin-1*β*
IL-4: Interleukin-4
IL-4R: Interleukin-4 receptor
IL-6: Interleukin-6
IL-10: Interleukin-10
NF-kB: Nuclear factor kappa B
LPS: Lipopolysaccharide
miRNA: MicroRNA
MRC-1: Mannose Receptor C, type 1
NOS2: Nitric oxide synthase 2
NOX2: NADPH oxidase 2
SOD1: Superoxide dismutase 1
TNF*α*: Tumor necrosis factor *α*.
